# Is there a place for measuring serum calcitonin prior to thyroidectomy in patients with a non-diagnostic thyroid nodule biopsy?

**DOI:** 10.20945/2359-3997000000320

**Published:** 2021-01-14

**Authors:** Diego Henrique Andrade de Oliveira, Luiz Pierre Huning, Mariana Comiran Belim, Patrick Fontes Rodrigues, Hildebrando Massahiro Nagai, Hans Graf

**Affiliations:** 1 Universidade Federal do Paraná Hospital de Clínicas Curitiba PR Brasil Serviço de Endocrinologia e Metabologia, Hospital de Clínicas, Universidade Federal do Paraná (SEMPR), Curitiba, PR, Brasil; 2 Universidade Estadual do Oeste do Paraná Cascavel PR Brasil Universidade Estadual do Oeste do Paraná (Unioeste), Cascavel, PR, Brasil; 3 União Oeste Paranaense de Estudos e Combate ao Câncer Cascavel PR Brasil União Oeste Paranaense de Estudos e Combate ao Câncer (Uopeccan), Cascavel, PR, Brasil

**Keywords:** Medullary thyroid cancer, fine-needle aspiration biopsy, calcitonin

## Abstract

**Objective:**

To verify the cytopathological Bethesda System classification of thyroid nodule fine-needle aspiration biopsy (FNAB) in MTC patients and to assess the role of preoperative serum calcitonin (CT) levels in the investigation of this neoplasm in medullary thyroid cancer (MTC) patients under observation at the Uopeccan (União Oeste Paranaense de Estudos e Combate ao Câncer).

**Materials and methods:**

This is a cross-sectional review of medical records of patients monitored at the thyroid cancer outpatient clinic of Uopeccan. Clinical and demographic data, laboratory tests, ultrasound images, and cytopathological findings of MTC patients were evaluated.

**Results and discussion:**

Among the 360 patients with thyroid cancer monitored in the outpatient clinic, 5.2% (n: 19/360) had MTC. The hereditary form was more prevalent (63.2%), and there was no sex preference. The most common ultrasound findings were hypoechogenicity, solid appearance and microcalcifications. The FNAB diagnoses showed a sensitivity of 47.1%, and the most common cytopathological report was Bethesda category III. Serum CT levels showed good sensitivity (84.6%) for the diagnosis of MTC, and sensitivity levels were directly associated with the size of the nodule and distant metastases.

**Conclusion:**

Bethesda category III was more prevalent in this group of MTC patients. Serum CT levels were more sensitive than cytopathology for diagnosis of this neoplasm and were able to identify all patients who could not be diagnosed by FNAB.

## INTRODUCTION

**M**edullary thyroid cancer (MTC) is a well-differentiated neuroendocrine carcinoma that affects thyroid C-cells. It represents approximately 5% of malignant thyroid nodules and is an aggressive neoplasm with an estimated 10-year survival rate of 40%-50% (1). MTC is responsible for up to 13% of all thyroid cancer deaths due to distant metastases (2).

MTC occurs in the sporadic form in 80% of cases, with the remaining 20% caused by an autosomal dominant genetic disorder associated with mutations in the *RET* gene (3). The distinction between these forms is of particular clinical relevance due to differences in prognosis and the need for family screening, genetic counseling, and follow-up for hereditary forms (4).

This neoplasm is difficult to diagnose prior to surgery. The few studies that analyzed ultrasound images of MTC patients found that the traditional risk factors used to characterize papillary thyroid carcinoma (PTC) are similar to those for MTC (5). Thus, the finding of a deeply hypoechoic nodule and the presence of gross intralesional calcifications may suggest MTC (6).

Fine-needle aspiration biopsy (FNAB) is a safe and useful procedure to detect MTC. Patients with this pathology who have been diagnosed by FNAB should undergo a careful preoperative assessment to determine the extent of the disease, analysis of the *RET* mutation, and, in cases of hereditary MTC, investigate associated pathologies, such as hyperparathyroidism (HPT) and pheochromocytoma (PHEO) (5).

**Figure 1:**
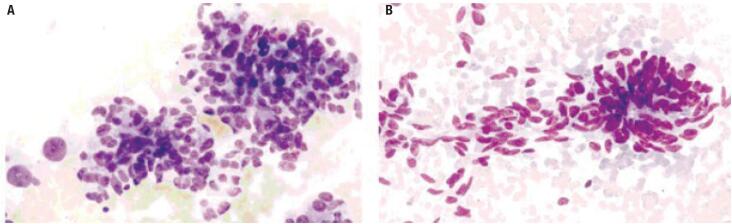
MTC cytological presentation examples in FNAB. (A) Bethesda III: Large clusters of polygonal and triangular cells. (B) Bethesda VI: abundant amyloid substance in the extracellular space with polygonal and triangular tumor cells.

Source: Adapted from Papaparaskeva and cols. (2000) (13).

However, the accuracy of cytopathology in detecting MTC is lower than in the case of PTC. In a meta-analysis that examined 15 papers, the accuracy of FNAB to diagnose MTC in patients with suspicious nodules was less than 50%. Part of this is due to the rarity of the disease and its multiple cytological manifestations (3). Notably, a previous study found that cytopathological examination classified up to 15% of patients as Bethesda category III, which is not conclusive (Figure 1) (7).

Molecular testing in indeterminate thyroid FNAB specimens could assist in the preoperative diagnosis of MTC (8). However, this method is expensive and not widely accessible in many medical centers.

Serum calcitonin (CT) measurements had a higher diagnostic sensitivity and specificity than FNAB analyses. Thus, CT is considered to be the main biochemical marker for diagnosis and postoperative follow-up of MTC patients due to its excellent sensitivity (95%), and because only 3%–10% of healthy patients have high serum CT levels. However, given that MTC is present in only 0.3%-1.4% of patients with thyroid nodules, expert opinions vary regarding the usefulness of routine serum CT measurement in this population (5).

It is very important to find feasible alternatives to increase the preoperative diagnostic accuracy of MTC since some aspects related to preoperative assessment differ from the common surgical approach for nodular thyroid disease. First, a preoperative diagnosis of PHEO mandates that adrenalectomy be performed prior to thyroidectomy (9). Second, total thyroidectomy (TT) should be performed with dissection of the bilateral central lymph node compartments due to the high rate of lymph node metastases in MTC patients. Finally, if primary HPT is diagnosed in the preoperative period of thyroidectomy, parathyroidectomy can be performed in the same surgical procedure (5).

The main objectives of this study were to verify the cytopathological Bethesda System classification of thyroid nodules FNABs in a group of MTC patients and to assess the role of preoperative serum CT levels in the investigation of this neoplasm. The secondary objectives were to estimate the diagnostic sensitivity of FNAB of the thyroid nodule among the study population, to describe clinical and demographic aspects of MTC patients, and to present the ultrasound characteristics of thyroid nodules diagnosed as MTC.

## MATERIALS AND METHODS

This is a quali-quantitative, documentary, and cross-sectional study conducted using data extracted from physical and electronic medical records of MTC patients assisted at the thyroid cancer outpatient clinic of Uopeccan (*União Oeste Paranaense de Estudos e Combate ao Câncer*) between 2013 and 2018.

This research was approved on May 25, 2018, by the Ethics Committee on Research with Human Beings, State University of Western Paraná (*Universidade Estadual do Oeste do Paraná*), Cascavel, Paraná, Brazil, registered on the Brazil Platform (*Plataforma Brasil*) under the protocol No. 89484218.7.0000.0107. The ethical committee waived the requirement of informed consent because this study consists of a review of information from medical records and the confidentiality of the information was maintained by the authors.

All patients diagnosed with MTC through analysis of the anatomopathological examination of the TT piece were included in the study. The exclusion criterion was the absence of an anatomopathological report confirming the MTC diagnosis of patients from other institutions who had an informal report of this neoplasm. Anatomopathological criteria reviewed by Wei (2020) were those considered to evaluate the TT piece (10).

Data sampling was performed using physical and electronic medical records from the TASY^®^ system at the Ciro Antonio Kreuz Study and Research Center, Uopeccan, between June and August 2018. All patients with thyroid cancer under follow-up at Uopeccan from 2013 to 2018 were considered for this study.

Clinical and demographic data, information on treatments performed and their complications, and information on the follow-up and evolution of patients with MTC were collected. The clinical and demographic data used were age, sex, age at diagnosis, family history of MTC, and occurrence of metastases. The ultrasound findings were reported using criteria from the American College of Radiologists (2017), such as echogenicity (very hypoechoic, hypoechoic, isoechoic, hyperechoic, or anechoic), composition (solid or almost completely solid, mixed cystic and solid, spongiform, or cystic), shape (wider than tall or taller than wide), margins (smooth, ill-defined, lobulated/irregular, or extra-thyroidal extension), and echogenic foci (none or large comet tall artifact, macro-calcifications, peripheral/rim calcifications, or punctate echogenic foci). Following ultrasound analysis, the characteristics of the nodules were scored to determine the TI-RADS grade, which can be categorized into TI-RADS 1 (benign), TI-RADS 2 (not suspicious), TI-RADS 3 (mildly suspicious), TI-RADS 4 (moderately suspicious), and TI-RADS 5 (highly suspicious). Finally, data were collected on the nodule vascularization pattern, number of nodules, and suspicious lymph nodes (11).

The cytopathological FNAB results of the thyroid nodule diagnosed with MTC were classified according to the Bethesda System, as follows: (I) non-diagnostic sample, (II) benign, (III) atypia of undetermined significance, (IV) suspicious for follicular neoplasm, (V) suspicious malignancy, and (VI) malignant. We considered diagnostic FNAB as those classified as Bethesda category VI or Bethesda category V with suspected MTC, following Suzuki and cols. (2017) (7).

The surgical methods performed and the anatomopathological data of the surgical products were analyzed, including the largest tumor diameter (cm), number of nodules, presence or absence of angiolymphatic infiltration, impairment of surgical margins, and presence of lymph node metastases.

During patient follow-up, pre- and postoperative serum CT levels and CT levels in the FNAB washouts were analyzed whenever such procedures were performed. These laboratory tests were analyzed by chemiluminescence, with normal values of CT < 8.4 pg/mL in men and CT < 5 pg/mL in women.

Imaging exams such as ultrasonography, tomography, magnetic resonance, and scintigraphy were performed to investigate local disease recurrence and/or distant metastases. *RET* gene sequencing was analyzed with the Sanger sequencing method using the BigDye Terminator and ABI 3130XL Genetic Analyzer (Thermo Fisher Scientific, Waltham, MA, USA). Due to the unavailability of *RET* sequencing at the time of diagnosis, this was done only after surgical treatment. Screening for PHEO and HPT before surgical treatment of MTC was performed to avoid surgical complications.

The data were collected as a single database in a Microsoft Excel spreadsheet and analyzed using descriptive statistics, including the arithmetic mean, standard deviation, median, minimum and maximum values, and frequencies. The correlation between nodule size and preoperative CT levels was calculated using Pearson’s correlation coefficient with the following reference values: 0 to 0.19, very weak correlation; 0.20 to 0.39, weak correlation; 0.40 to 0.69, moderate correlation; 0.70 to 0.89, strong correlation; 0.90 to 1, very strong correlation. Where continuous variables had a parametric distribution, they were analyzed using the Student’s t-test, while non-parametric variables were analyzed with the Mann-Whitney test and Welch’s t-test. The Student’s t-test was chosen when the hypothesis of normality and homoscedasticity of the data were verified and the Welch’s t-test was selected when the hypothesis of normality was verified but the hypothesis of homoscedasticity was not, while the Mann-Whitney test was chosen when the data were not normally distributed. Categorical variables were analyzed with the chi-square test (χ^2^ test) when all the necessary conditions were met, while Fisher’s exact test was performed for cases that did not meet the necessary criteria. Results with p-values < 0.05 were considered as statistically significant. IBM SPSS Statistics version 25 - © 1989 - 2017 was used to perform the statistical tests.

## RESULTS

We identified 360 patients with thyroid cancer who were under follow-up at the Uopeccan outpatient clinic. A total of 19 patients (5.2% of the studied population) were diagnosed with MTC; 12 of these patients had hereditary MTC (Table 1) and were referred to our clinic after the index case of the family had started treatment at Uopeccan. The mean age of MTC patients was 44.5 (±9.1) years in the sporadic form, with female patients predominating in this subgroup (71.5%). The mean age of patients with the hereditary form was 30.7 (±15.5) years, with male patients more common (58.3%). The mean follow-up time of all patients at Uopeccan was 4.42 (±3.77) years, ranging between one month and 13 years.

**Table 1:** Demographic, clinical and ultrasound characteristics of MTC patients

		Sporadic form (n = 7)	Hereditary form (n = 12)	All patients (n = 19)
Mean age (years)		44.5 (±9.1)	30.7 (±15.5)	35.8 (±14.8)
Sex				
	Male	28.5% (2)	58.3% (7)	47.3% (9)
	Female	71.5% (5)	41.7% (5)	52.7% (10)
Ultrasound characteristics*				
	Hypoechogenic	100% (6)	100% (10)	100% (16)
	Solid appearance	66.7% (4)	80% (8)	75% (12)
	Mixed cystic and solid appearance	33.3% (2)	20% (2)	25% (4)
	Irregular margin	50% (3)	30% (3)	37.5% (6)
	Presence of microcalcifications	50% (3)	80% (8)	68.75% (11)
	Shade (wider than tall)	100% (6)	100% (10)	100% (16)
	Suspicious lymph nodes	50% (3)	20% (2)	31.25% (5)
	Multinodular goiter	66.7% (4)	70% (7)	68.75% (11)
TI-RADS*				
	TR1 or TR2	0% (0)	0% (0)	0% (0)
	TR3	16.6% (1)	0%	6.25% (1)
	TR4	50% (3)	80% (8)	68.75% (11)
	TR5	33.3% (2)	20% (2)	25% (4)

Frequencies are represented in percentage, and absolute numbers are in parentheses.

* Three patients were excluded from the analysis of the ultrasound findings due to the lack of a preoperative cervical ultrasound report.

Source: the authors.

Hypoechogenicity (100%), solid appearance (75%), microcalcifications (68,75%), and multinodular disease (68.75%) were the most frequent ultrasound findings. TI-RADS 4 and TI-RADS 5 represented 93.75% of the reports among patients who underwent an ultrasound (Table 1).

The results of FNAB, according to the Bethesda System for thyroid cytopathological reports, are presented in Table 2. FNAB was performed in 17 patients preoperatively, with the most common result being Bethesda category III (35.3%). Bethesda category II was observed in three patients (17.65%), of whom two had the sporadic form. One of these patients underwent thyroidectomy due to compressive symptoms of the goiter and had an incidental medullary microcarcinoma in the proximity of a colloid nodule. The other individual with sporadic MTC had been submitted to two FNABs within an interval of approximately nine months, which resulted in a Bethesda II lesion. This patient underwent thyroidectomy due to suspicious echographic findings (TI-RADS 5) and a 1.8 cm MTC was found during the anatomopathological exam.

**Table 2:** Cytopathological Bethesda system classification of thyroid nodule and clinical, laboratory, and anatomopathological findings of MTC patients

		Sporadic form (n = 7)	Hereditary form (n = 12)	All patients (n = 19)
Bethesda category of suspicious nodule*				
	I	0 (0)	0 (0)	0 (0)
	II	33.3% (2)	9% (1)	17.6% (3)
	III	16.6% (1)	45.4% (5)	35.3% (6)
	V	33.3% (2)	18.1% (2)	23.5% (4)
	VI	16.6% (1)	27.2 % (3)	23.5% (4)
Preoperative CT levels (pg/mL)**				
	Median and range	2703 (1407-4000)	509 (14.9-5620)	629 (14.9-5620)
Nodule size (cm)***				
	Median and range	2 (1-7.4)	1.85 (0.4-7.5)	1.85 (0.4-7.5)
Central compartment involvement		42.9% (3)	33.3% (4)	36.8% (7)
Lateral compartment involvement		42.9% (3)	33.3% (4)	36.8% (7)
Distant metastases		28.6% (2)	41.7% (5)	36.8% (7)

CT: calcitonin.

The frequencies are represented in percentage and absolute numbers are in parentheses for the Bethesda category of suspicious nodule, distant metastases and involvement of the central and lateral compartments. Calcitonin levels and nodule size are expressed as medians and range.

* Two patients were excluded from Bethesda cytopathological analysis due to the lack of a FNAB report.

** Seven patients were excluded from the analysis of CT levels due to the lack of serum CT data.

*** One patient was excluded from the nodule size analysis because they did not have an adequate anatomopathological description. Source: the authors.

A thirteen-year-old female patient from a family with hereditary MTC was classified as Bethesda category II by FNAB of a 0.5 cm nodule and serum CT value was 8 pg/mL (normal < 5 pg/mL). She had a suspected preoperative diagnosis of MTC after dosing CT levels in the FNAB washout two months later, which gave a positive result with CT in the FNAB washout of over 5,000 pg/mL with a serum CT of 100 pg/mL.

Reports of Bethesda categories V (suspicious for MTC) and VI, termed diagnostic FNAB in this study, had a sensitivity of 47.1% for MTC. No patient with Bethesda category V was suspected of having another subtype of thyroid cancer.

Preoperative serum CT levels were measured in 13 patients, including one patient described above with Bethesda II lesion and four of the six patients who were classified into Bethesda category III since they showed high clinical suspicion for MTC due to their family history. Sequencing of the *RET* gene was not available at that time. The median preoperative serum CT was 2703 pg/mL (range 1407-4000 pg/mL) among patients with the sporadic form and 509 pg/mL (range 14.9-5620 pg/mL) in patients with the hereditary form (Table 2).

Serum CT levels were elevated in all patients, but two patients with the hereditary form with nodules between 0.4 and 0.5 cm had preoperative serum CT levels less than twice the upper limit of normal. The remaining patients had markedly elevated serum CT (greater than 100 pg/mL). If the patients with slightly elevated preoperative serum CT are disregarded, the diagnostic sensitivity of elevated serum CT for MTC is 84.6%. Elevated serum CT was significantly correlated with a diagnostic FNAB (p < 0.05) and its quantitative value was significantly correlated with the risk of malignancy calculated from cytopathological analysis.

**Figure 2:**
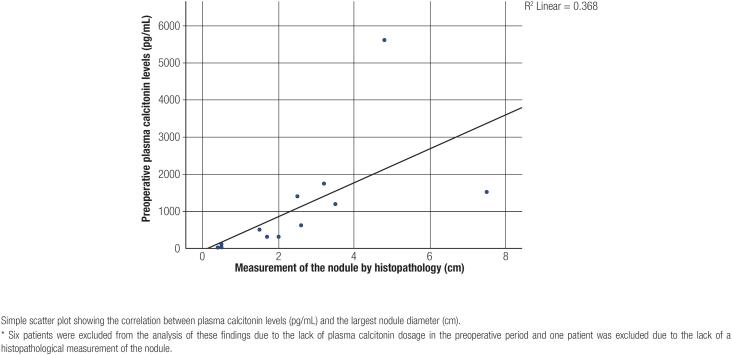
Scatter plot with an adjustment line for preoperative serum calcitonin levels vs. nodule diameter.

Source: the authors.

The median size of the nodules at anatomopathological examination was 1.85 cm, with the smallest nodule measuring 0.4 cm and the largest 7.5 cm (Table 2). The correlation between preoperative CT levels and nodule size was statistically significant (p < 0.05), with Pearson’s index showing a moderate positive correlation (0.606) (Figure 2).

The hereditary form of MTC was identified in 63.2% of the patients (n = 12) with a positive diagnosis. Of these, six patients received a diagnosis of PHEO during the preoperative period of TT or up to one year afterward, and five patients had diagnostic FNAB and/or high preoperative CT levels. Adrenalectomy prior to TT was performed in four patients. A case from another institution with a diagnostic FNAB classified as Bethesda category V was submitted to adrenalectomy seven months after TT. Another patient with diagnostic FNAB classified as Bethesda category III underwent adrenalectomy five months after TT in our institution. In three families, the hereditary form was confirmed during follow-up through the sequencing of the *RET* gene. Mutations in codon 634 (NEM 2A) were identified in two families (with nine and two cases, respectively), and codon 918 (NEM 2B) was identified in one patient.

**Figure 3:**
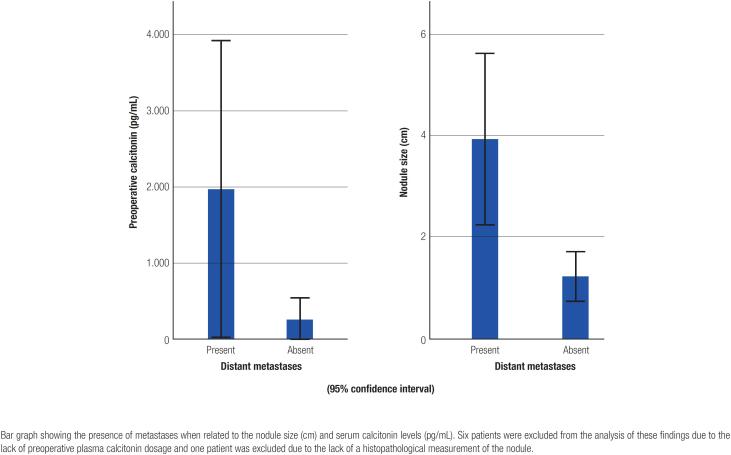
Simple bars showing the presence of distant metastases vs. preoperative serum calcitonin levels and distant metastases vs. nodule size.

Source: the authors.

Lymph node metastases in the central and lateral compartments and distant metastases occurred in 36.8% (n = 7) of all patients. Preoperative CT levels and nodule size were significantly associated with distant metastases (Figure 3).

## DISCUSSION

MTC is an aggressive disease that is rare among malignant thyroid neoplasms. Magalhães and cols. (2003) observed that 3%-10% of all thyroid cancer cases are MTC with no sex preference (4). In our outpatient clinic, we found a frequency of 5.2%, also with no sex preference (52.7% were female). Overall, the sporadic form is considered the most frequent. For example, in a manuscript published by Pacini and cols. (2010), 70-80% of cases were sporadic (12). On the other hand, in our sample, the hereditary form was the most prevalent (63.2%). This may be due to the clinical suspicion of hereditary MTC in patients at our clinic and an active search among their relatives.

MTC is challenging to diagnose preoperatively. According to Trimboli and cols. (2014), although cervical ultrasound is not specific for the diagnosis of this neoplasm, the latter can exhibit signs such as hypoechogenicity and microcalcifications, which suggest a higher risk of malignancy (6). All patients diagnosed with MTC at the anatomopathological examination exhibited hypoechogenicity, with microcalcifications identified in about 2/3 of them. These were the main ultrasound findings that allowed the radiologist to classify these patients as TI-RADS 4 and TI-RADS 5.

FNAB has a high diagnostic value for PTC. However, is sensitivity for MTC is low. Although older studies like Papaparaskeva and cols. (2000) reported sensitivity rates of nearly 89% in 91 MTC lesions (13), a more recent meta-analysis of 15 studies with 641 MTC lesions conducted by Trimboli and cols. (2015) reported a sensitivity value of 56.4% for MTC (3), which was consistent with our findings (47.1%).

Another study published by Essig and cols. (2013) described the following diagnoses of 245 sporadic MTC patients: not adequate, 6.12%; benign, 6.12%; indeterminate, 26.12%; other malignancies, 15.92%; MTC or suspected MTC, 45.71% (14). Similar results were observed in our group of patients, as follows: benign, 17.6%; atypia of undetermined significance, 35.3%; malignant or suspected MTC, 47.1%. The high frequency of patients classified as Bethesda category II may be due to the presence of small nodules (< 1 cm) in two of the three patients. It is possible that the material collected in the FNAB is from the adjacent thyroid parenchyma.

Due to the low sensitivity of FNAB in providing a diagnostic hypothesis of Bethesda V or Bethesda VI for MTC, other methods to investigate this neoplasm should be used to increase sensitivity when a FNAB is not diagnostic. Allelein and cols. (2018) reported that serum CT had a diagnostic sensitivity of 93% for MTC (15), which our study corroborates (84.6%).

The low cure rate once the disease spreads beyond the thyroid gland supports the use of serum CT screening in the early diagnosis of MTC in patients with thyroid nodules. However, given that MTC is present in only 0.3%-1.4% of patients with thyroid nodules, issues of cost effectiveness have been raised when considering routine serum CT measurement in this population. Additionally, the clinical significance and natural history of MTC diagnosed by CT screening is unknown. Although this practice is the standard of care at some centers in European countries, there has been controversy around its application in the United States (5).

This is a subject of much discussion among the ATA task force, which further highlights the lack of consensus among specialists regarding the cost effectiveness of the measurement of serum CT in thyroid nodular disease, with most clinics not observing the practice (5). However, an area for further discussion is the measurement of serum CT prior to thyroidectomy when deciding on surgical treatment due to successive FNABs with undetermined cytology (especially when there is no availability of molecular markers) or in the event of ultrasound findings that are highly suggestive of malignancy despite benign FNAB findings.

These findings highlight the importance of providing greater perioperative safety to patients who will definitely undergo a thyroid operation due to the conditions described above. Doing so decreases the probability of becoming aware of MTC only after the operation has been completed. Even though MTC is an infrequent diagnosis among Bethesda III patients diagnosed with FNAB for nodular diseases in general, as reported by Heller (2014), the preoperative diagnostic hypothesis of MTC may lead to an investigation of related neoplasms and may alter the extent of the procedure (16). We document a high sensitivity of plasma CT levels in patients with MTC who were diagnosed with Bethesda category III by FNAB, all of whom had high levels.

The possibility of a preoperative diagnosis of MTC can reduce the surgical morbidity of a second operation for the lymph node dissection of the recurrent chain, in addition to ensuring more safety in the operative act by excluding the presence of concomitant PHEO. We highlight the importance of diagnosing PHEO prior to thyroidectomy. Two of our patients received the diagnosis of PHEO only in the early postoperative period of TT, which put their lives at risk, since performing TT in patients with undiagnosed PHEO increases morbidity and the chance of perioperative death (9).

The size of the nodules diagnosed as MTC during the anatomopathological examination of the thyroidectomy products of our patients was correlated positively with the preoperative CT levels. These results are in line with those of Cohen and cols. (2000), who observed that larger tumors were associated with higher serum CT levels due to C-cell proliferation (17). On the other hand, MTC can also be found in patients with borderline or even normal serum CT levels. We followed a case of an individual with clinical suspicion of hereditary MTC with a TIRADS 4 micronodule (less than 0.5 cm) that was classified as Bethesda II by the FNAB and showed serum CT in the border zone. This patient had a preoperative diagnosis of MTC suggested by the CT levels in the FNAB washout since we were unable to perform the genetic test at that time.

Quantitative values of serum CT were higher in patients with diagnostic FNAB than in those with non-diagnostic FNAB (Bethesda II and III). Although we did not find a similar result in the literature, more advanced cases of the disease have been found to be associated with higher values of serum CT and larger numbers of histological alterations. Additionally, the levels of serum CT and size of nodules are directly associated with the number of metastases, as previously described in the literature (5).

Distant metastases of MTC occur in about 10% of patients (5,18). However, we found a higher rate (36.84%) of distant metastases, likely to be related to late diagnosis due to the patients’ difficulty in reaching a specialized oncology center.

The main limitations of our study are the retrospective model, the small sample size, and the possible overestimated sensitivity of serum CT levels since we measured it only in patients who previously had an indicative factor for MTC. Nevertheless, this study has internal validity. For this group of patients, the most frequent cytopathological finding was Bethesda category III, and the level of plasma CT could increase the preoperative diagnostic sensitivity of MTC.

Other centers specialized in thyroid cancer should develop similar studies to more robustly assess the recommendation of measuring preoperative serum CT levels in patients that will undergo thyroidectomy due to suspected lesions for malignancy verified by ultrasound or cytopathological examination resulting in Bethesda category III. Furthermore, studies including a large cohort that analyzes all patients with a thyroid nodule and a Bethesda III and IV cytology, independent of the final pathology, should be performed to confirm the role of serum calcitonin in this situation.

In conclusion, MTC was prevalent in 5.2% of patients being monitored for thyroid carcinoma in the Uopeccan outpatient clinic. The hereditary form was more prevalent, and there was no sex preference. The most common ultrasound findings were hypoechogenicity, solid appearance, and microcalcifications but they are not specific for the diagnosis of MTC. The most frequent cytopathological finding was Bethesda category III, and the sensitivity of diagnostic FNAB was low, consistent with earlier studies.

Serum CT levels were more sensitive than cytopathology for the MTC diagnosis and helped us to identify all patients not diagnosed by FNAB in our series of MTC patients. We believe that measuring serum CT levels in the preoperative period is an ideal tool to increase the perioperative safety of thyroidectomy. This measurement could be indicated when successive FNABs show undetermined cytology or ultrasound findings that are very suggestive of malignancy, despite benign FNAB findings. Additional studies are needed to strengthen the indication of measuring serum CT prior to thyroidectomy in these situations.
